# The Response of Maize to Inoculation with *Arthrobacter* sp. and *Bacillus* sp. in Phosphorus-Deficient, Salinity-Affected Soil

**DOI:** 10.3390/microorganisms8071005

**Published:** 2020-07-04

**Authors:** Tchuisseu Tchakounté Gylaine Vanissa, Beatrice Berger, Sascha Patz, Matthias Becker, Veronika Turečková, Ondřej Novák, Danuše Tarkowská, Fankem Henri, Silke Ruppel

**Affiliations:** 1 Leibniz Institute of Vegetable and Ornamental Crops Grossbeeren, Theodor- Echtermeyer-Weg 1, 14979 Grossbeeren, Germany; ruppel@igzev.de; 2 Department of Plant Biology, Faculty of Sciences, University of Douala, P.O. Box 24157 Douala, Cameroon; fankemhenri@yahoo.fr; 3 Faculty of Life sciences Humboldt-University of Berlin, Invalidenstraße 42, 10115 Berlin, Germany; 4 Institute for National and International Plant Health, Julius Kuehn-Institute—Federal Research Centre for Cultivated Plants, Messeweg 11/12, 38104 Braunschweig, Germany; beatrice.berger@julius-kuehn.de (B.B.); matthias.becker@julius-kuehn.de (M.B.); 5 Algorithms in Bioinformatics, Center for Bioinformatics, University of Tuebingen, Sand 14, 72076 Tuebingen, Germany; sascha.patz@uni-tuebingen.de; 6 Laboratory of Growth Regulators, The Czech Academy of Sciences, Institute of Experimental Botany and Palacky University, Slechtitelu 27, CZ-783 71 Olomouc, Czech Republic; veronika.tureckova@upol.cz (V.T.); novako@ueb.cas.cz (O.N.); tarkowska@ueb.cas.cz (D.T.)

**Keywords:** bacterial inoculation, P deficiency, salinity stress, maize, plant growth, phytohormone production

## Abstract

Salinity and phosphorus (P) deficiency are among the most serious soil factors constraining crop productivity. A proposed strategy for alleviating these stresses is supporting plants by inoculation with growth-promoting rhizobacteria (PGPR). Here, a comparison of the ability of two maize composite and two F1 hybrid varieties to tolerate a P deficiency in either a saline or a non-saline environment showed that the uptake of nutrients by all four entries was significantly reduced by the imposition of both soil salinity and P deficiency, and that their growth was compromised to a similar extent. Subsequently, the ameliorative effect of inoculation with three strains of either *Arthrobacter* sp. or *Bacillus* sp. in an environment, which suffered simultaneously from salinity and P deficiency, was investigated. Inoculation with each of the strains was found to limit the plants’ uptake of sodium cations, to increase their uptake of potassium cations, and to enhance their growth. The extent of the growth stimulation was more pronounced for the composite varieties than for the F1 hybrid ones, although the amount of biomass accumulated by the latter, whether the plants had been inoculated or not, was greater than that of the former varieties. When the bacterial strains were cultured in vitro, each of them was shown as able to produce the phytohormones auxin, abscisic acid, gibberellins, and cytokinins. The implication is that since the presence in the rhizospere of both *Arthrobacter* sp. and *Bacillus* sp. strains can support the growth of maize in salinity-affected and P deficient soils in a genotype-dependent fashion, it is important to not only optimize the PGPR strain used for inoculation, but also to select maize varieties which can benefit most strongly from an association with these bacteria.

## 1. Introduction

Salinity and phosphorus (P) deficiency represent two of the most serious soil constraints to crop production. Maize (*Zea mays*), a key subsistence crop throughout Latin America and Africa, is sensitive to salinity and its growth is compromised by P deficiency [1]. Tolerance to salinity requires the plant both to be able to maintain its uptake of water despite the osmotic pressure exerted by the presence of salt in the soil water, and the means to ensure that the cytosol retains a favorable K^+^/Na^+^ ratio [2]. Plants typically attempt to adapt to P deficiency by extending their roots, by increasing lateral root density, and by developing more root hairs [2]. The mean yield of maize in Cameroon is ~2.1 t/ha, a level which represents only 19% of what is attained by some producers in developed countries [3]. The crop’s relatively poor performance in the Cameroon, at least in part, is due to the fact that salinity affects about 671 thousand hectare of arable land [4], while some 85% of the soil is acidic and therefore P deficient [5]. While breeding for adaptation to either saline and/or P deficient soils has the potential to raise yield potential [6], the yield can also be enhanced by exploiting the ability of plant growth-promoting rhizobacteria (PGPR) to solubilize phosphate in the soil and to mitigate salinity stress [7]. Some of these bacteria are also known to produce a number of key phytohormones, notably auxin (indole-3-acetic acid, IAA), cytokinins (CKs), abscisic acid (ABA), and gibberellins (GAs), to convert atmospheric nitrogen into a form assimilable by plants and to enhance tolerance against certain pests [8].

Halotolerant phosphate solubilizing bacteria have recently attracted the attention of researchers engaged in efforts to improve crop performance in salinity-affected and P deficient soils. These bacteria belong to various genera, most notably *Bacillus* and *Arthrobacter*; while their ability to alleviate either salinity stress or P deficiency has been documented [5], their capacity to protect maize plants exposed simultaneously to these two stress agents has not been explored to date. Strains of both *Arthrobacter* sp. and *Bacillus* sp. have been shown to boost the growth of groundnut (*Arachis hypogaea*) in the face in salinity-affected and P deficient soils as a result of their ability to solubilize phosphate [7]. However, it has been suggested that PGPR species which have a positive effect on the growth of one plant species may not be equally effective for other plant species, and some bacteria colonize either just one single or a limited range of hosts [9]. Gaining an understanding of the basis of this species-dependency has been recognized as key to improving the level and reliability of plant growth stimulation contributed by PGPR [10].

Three *Arthrobacter* sp. (V54, V64, and V84) and three *Bacillus* sp. (V62, V39, and V1) strains have been successfully isolated from the rhizosphere of maize plants grown in Cameroon [11]. Each was associated with an individual set of plant growth-promoting traits, including the ability to solubilize rock phosphate, to fix atmospheric nitrogen, to produce siderophores, to tolerate salt, to encourage the germination of maize, and to support the growth of maize plants [11]. Further experiments have shown them to be also capable of solubilizing phosphate under saline conditions (unpublished data). The present study focused on the response of four (two composite and two F1 hybrid varieties) widely grown, local maize varieties to previously selected *Arthrobacter* sp. and *Bacillus* sp. strains under combined salinity and P stress condition. We aimed to find the best positive bacterial strain-maize cultivar interaction for further improvement of plant growth promotion under combined salinity and P stress condition and prove the plant cultivar dependent interaction between selected efficient native PGPR and maize composites and hybrid varieties. Hence, we hypothesized that: (i) Maize cultivars will respond differently to a combined P deficiency and salinity stress condition; (ii) inoculation of PGP strains will reduce the detrimental effect of salinity and P stress on plants and the bacterial plant growth-promoting effect on composite and hybrid maize varieties will be different. The main purpose was to assess the potential of the six PGPR strains to mitigate the growth inhibition imposed by the combined salinity stress and P deficiency.

## 2. Materials and Methods

### 2.1. Inoculum Preparation

Inoculum was prepared from each bacterial strain by transferring a single colony into a 50 mL standard nutrient broth I (Carl Roth, Karlsruhe, Germany) and shaking at 180 rpm for 24 h with the temperature held at 28 °C. The cells were harvested by centrifugation (10,000 x *g,* 15 min, 4 °C), rinsed three times in sterile 0.05 M NaCl, and then suspended in sufficient 0.05 M NaCl to give an OD_620_ of 0.2 for the in vitro experiments and to achieve a concentration of 10^8^ colony forming units per mL for the greenhouse experiments.

### 2.2. Plant Material and Culture

The four maize varieties tested comprised the Cameroonian composite varieties (Cameroon Maize Series: CMS 8704 and CMS 8501), and the two Cameroon highland F1 hybrid varieties (CHH 101 and CHH 103). These varieties provided by the Agricultural Research Institute for Development (IRAD) were chosen for being among the most widely composite and hybrid maize varieties, respectively, that were grown in the Cameroon. The key characteristics of all four varieties are detailed in Table 1.

For the greenhouse experiment designed to assess tolerance to salinity stress and/or P deficiency, grains were surface-sterilized by immersion for 10 min in 4% v/v sodium hypochlorite, rinsed five times in sterile distilled water, and allowed to germinate for 14 days by planting in sand in a chamber delivering a 24 h photoperiod and a day/night temperature regime of 25/20 °C. Each 14-day old plant was potted into 1 L of a 1:1 mixture of sand and vermiculite, and the pots were placed in a greenhouse delivering a day/night temperature regime of 25/23 °C and 75% relative humidity.

Four treatments were imposed: Treatment #1 (control), in which the plants were supplied with Hoagland’s nutrient solution containing soluble phosphate (KH_2_PO_4_, equivalent to 80 kg P ha^−1^), EC of 0 ds m^−1^; treatment #2 (salinity stress), in which the treatment #1 solution was supplemented with sufficient NaCl to generate an EC of 12 ds m^−1^; treatment #3 (P deficiency), in which the plants were provided with Hoagland’s solution lacking soluble phosphate but with added rock phosphate (RP) in pots (350 mg P g^−1^ soil) EC of 0 ds m^−1^; and treatment #4 (combined salinity stress and P deficiency), in which the medium contained both RP and NaCl (EC of 12 ds m^−1^). Treatment #1 plants were watered five times per week with 30 mL of the nutrient solution and twice a week with 30 mL reverse osmosis water. Before mixing in treatments #3 and #4 pots, the RP was rinsed four times with warm water in the following cycle: 1 h-2 h–1 h-24 h, dehydrated by baking at 60 °C, and ground to a powder. For treatments #3 and #4, K_2_SO_4_ was added to ensure that the same K concentration pertained across all four treatments. Each of the treatments was applied to six pots of each of the four varieties, and the pots were arranged into completely randomized blocks. The plants were assessed six weeks after transplanting in the greenhouse.

### 2.3. Assessment of Plant Performance

At harvest, the lengths of both the longest root and the stem were recorded. Shoot and root fresh weights were determined, and the samples were reweighed after baking at 60 °C for 72 h to determine dry weights. To assess P, K and Na contents using optical emission spectrometry and inductively coupled plasma (ICP-OES), the oven-dried root and stem samples were pulverized in a Pulverisette plant grinder (Fritsch, Oberstein, Germany) and digested following an established protocol [12], in which a 0.5 g aliquot was mixed with 65% HNO_3_ and 30% v/v hydrogen peroxide within a MARS 5 Xpress pressure digestion system (CEM GmbH, Kamp-Lintfort, Germany). The elements were extracted using a defined temperature program. The contents of P, K, and Na were estimated spectrophotometrically, using wavelengths of, respectively, 178.28 nm (axial observation), 766.49 nm (radial observation), and 589.59 nm (radial observation) by an iCAP 7400 device (Thermo Fisher Scientific GmbH Dreieich, Germany).

### 2.4. The Effect of Bacterial Inoculation on Maize Plants Exposed to Salinity Stress and P Deficiency

The efficacy, with respect to plant growth and nutrient uptake, of six bacterial strains (*Arthrobacter* sp. strains V54, V64, V84 and *Bacillus* sp. strains V62, V39, V1) was tested on all four maize varieties. Surface-sterilized grains were immersed in either one of the bacterial suspensions or in 0.05 M NaCl for 15 min, and then allowed to germinate for 14 days by planting in sand in a chamber delivering a 24 h photoperiod and a day/night temperature regime of 25/20 °C. Each plant was potted into 1 L of a 1:1 mixture of sand and vermiculite, and the pots were placed in a greenhouse providing a day/night temperature regime of 25/23 °C and 75% relative humidity. The plants were all subjected to treatment #4 (see above) for six weeks. One day after potting, 2.5 mL of the bacterial suspension (or, for the controls, 2.5 mL 0.05 M NaCl) was given to each pot. The experiment was set out as completely randomized blocks involving seven treatments (six bacterial inoculations and the control), replicated six times for each of the four varieties. Post-harvest procedures were as described above.

### 2.5. Phytohormone Analysis

To quantify the production by the bacteria of phytohormone, a 1 mL aliquot of each bacterial suspension (OD_620_ = 0.2) was inoculated into 50 mL of a standard nutrient broth I containing 1 g L^−1^ of L-tryptophan. The bacteria were incubated in glass vials for 48 h at 28 °C with continuous shaking, and harvested by centrifugation (10,000× *g*, 15 min, 4 °C) for the next steps.

#### 2.5.1. Measurements of Auxins (IAA) and Cytokinins

The content of CKs, auxin (IAA and its catabolite 2-oxindole-3-acetic acid (oxIAA)) was determined using LC-MS/MS [13,14]. A ~5 mg aliquot of lyophilized bacterial cells was homogenized and extracted in 1 mL of 60% v/v methanol, 10% formic acid, and 30% H_2_O. Alternatively, 1 mL of the liquid culture was diluted by the addition of 10 mL of the above extraction solution. A mixture of stable, isotope-labeled internal standards (0.25 pmol CK bases, ribosides, *N*-glucosides, 0.5 pmol CK *O*-glucosides and nucleotides, 5 pmol ^13^C_6_-IAA, and ^13^C_6_-oxIAA per sample) was added to each sample for the purpose of validation [13]. The extracts were first passed through an octadecylsilica-based column (C18, 500 mg of sorbent) (Applied Separations, Inc., Allentown, PA, USA) then through an Oasis MCX column (30 mg mL^−1^) (Waters Corp., Milford, MA, USA) [15]. The analytes were eluted using a three-step procedure involving 60% v/v methanol, 0.35 M aqueous NH_4_OH, and 0.35 M NH_4_OH in 60% v/v methanol. Two alkaline eluates containing CK metabolites were further purified by immunoaffinity extraction [16]. The levels of CKs, free IAA, and oxIAA were determined using an UHPLC-ESI (−/+)-MS/MS system, in which an Acquity UPLC I-Class System device was coupled to a Xevo TQ-S MS device (Waters Corp.). Stable isotope-labelled internal standards were included for reference.

#### 2.5.2. Determination of Abscisic Acid (ABA) and Gibberellins (GAs)

To determine the content of ABA using ultra performance liquid chromatography-electrospray-mass spectrometry (UHPLC-ESI (−/+)-MS/MS), an aliquot of bacterial suspension containing 5 mg cells were shaken in an MM301 vibration mill (Retsch GmbH & Co. KG, Haan, Germany) at 27 Hz for 3 min in the presence of 3 mm diameter tungsten carbide beads. An internal standard containing 20 pmoL (+)-3’,5´,5´,7´,7´,7´-^2^H_6_-ABA (OlchemIm, Olomouc, Czech Republic) and 1 mL ice-cold methanol/water/acetic acid (10/89/1, v/v) were added to each of the samples. After a 1 h period of shaking in the dark at 4 °C, the preparations were centrifuged (20,000× *g*, 10 min, 4 °C) and the pellets re-extracted in a 0.5 mL extraction solvent for 30 min. The combined extracts were purified by passage through an Oasis® HLB cartridge (60 mg, 3 mL) (Waters Corp.), then evaporated to dryness in a Speed-Vac (UniEquip, Planegg, Germany), and finally analyzed by UHPLC-ESI (−/+)-MS/MS [17].

The sample preparation and analysis of GAs was performed according to the method described in Urbanová et al. [18] with some modifications. Liquid bacterial media were prepared for ultra-trace analysis of GAs as follows: 5 mL of 100% acetonitrile was added to 5 mL of bacterial media (protein precipitation) with addition of 17 internal GAs standards ([^2^H_2_]GA_1_, [^2^H_2_]GA_3_, [^2^H_2_]GA_4_, [^2^H_2_]GA_5_, [^2^H_2_]GA_6_, [^2^H_2_]GA_7_, [^2^H_2_]GA_8_, [^2^H_2_]GA_9_, [^2^H_2_]GA_15_, [^2^H_2_]GA_19_, [^2^H_2_]GA_20_, [^2^H_2_]GA_24_, [^2^H_2_]GA_29_, [^2^H_2_]GA_34_, [^2^H_2_]GA_44_, [^2^H2]GA51, and [^2^H_2_]GA_53_; purchased from OlChemIm, Czech Republic) and incubated at −20 °C for 60 min. The samples were then centrifuged at 3180 rcf and 4 °C for 20 min. The supernatant was removed and evaporated to dryness in vacuo. The sample residue after evaporation was extracted with 1 mL of ice-cold 80% acetonitrile containing 5% formic acid and samples were further purified using reversed-phase and mixed mode SPE cartridges (Waters, Milford, MA, USA) and analyzed by ultra-high performance liquid chromatography-tandem mass spectrometry (UHPLC-MS/MS; Micromass, Manchester, UK). GAs were detected using a multiple-reaction monitoring mode of the transition of the ion [M–H]^−^ to the appropriate product ion. The Masslynx 4.1 software (Waters, Milford, MA, USA) was used to analyze the data and the standard isotope dilution method was used to quantify the GAs levels [19].

### 2.6. Statistical Analysis

All data were subjected to a standard analysis of variance. Treatment means were compared using Tukey’s HSD test, with the significance threshold set at 0.05. All necessary calculations were performed using the Sigma Plot software v12.3 (www.sigmaplot.co.uk).

## 3. Results

### 3.1. The Stress Response of the Four Maize Varieties

**Figure 1:**
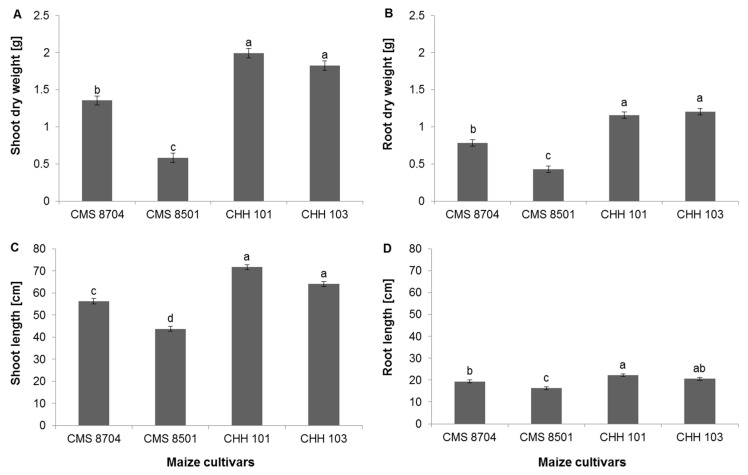
The accumulation of biomass by eight-week-old plants, averaged over all four treatments (no stress, salinity stress, phosphorous (P) deficiency, and salinity stress + P deficiency). (**A**) Shoot dry weight, (**B**) root dry weight, (**C**) shoot length, (**D**) root length. CMS 8704, CMS 8501: Composite varieties, CHH 101, CHH 103: F1 hybrid varieties. Values are shown in the form mean ± SD (*n* = 6). Within each variety, values marked by the same letter do not differ significantly (*p <* 0.05) from one another.

Averaged over all four treatments, both the shoot and root growth of the two hybrid varieties was superior to those of the two composite varieties (Figure 1 and Figure S1). While the performance of the hybrid varieties was quite similar to one another, the composite variety CMS 8501 plant shoot and root growth was less robust than that of CMS 8704 plants (Figure 1).

**Figure 2:**
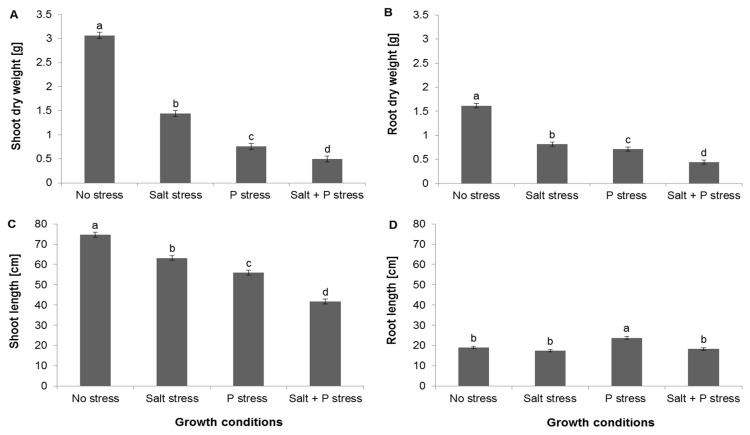
The depressive effect on the growth of four test maize varieties (CMS 8704, CMS 8501: Composite varieties, CHH 101, CHH 103: F1 hybrid varieties) of salinity stress, P deficiency, and salinity stress + P deficiency. The histogram columns represent the mean effect, averaged over all four maize varieties, of each stress treatment on (**A**) shoot dry weight, (**B**) root dry weight, (**C**) shoot length, (**D**) root length. Values are shown in the form mean ± SD (*n* = 6). Values marked by the same letter do not differ significantly (*p <* 0.05) from one another.

Exposure to salt stress compromised both shoot elongation and the accumulation of biomass by both the shoot and root more severely than exposure to P deficiency, while exposure to combined salt and P stress was even more damaging (Figure 2). Root elongation was not inhibited by neither salinity stress or combined P and salt stress treatments and was even promoted by P deficiency.

**Figure 3:**
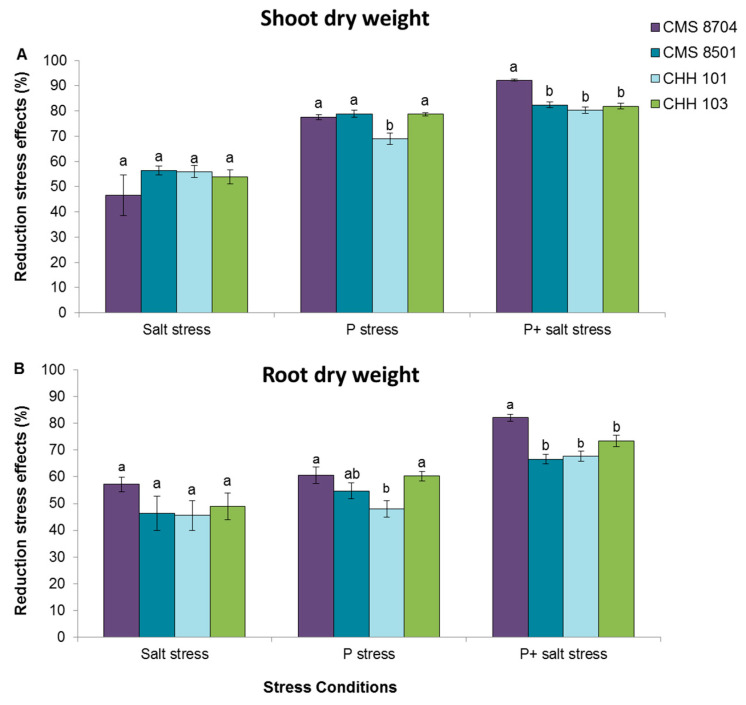
The depressive effect on the growth of four test maize varieties (CMS 8704, CMS 8501: Composite varieties, CHH 101, CHH 103: F1 hybrid varieties) of salinity stress, P deficiency, and salinity stress + P deficiency as reflected by (**A**) shoot dry weight, (**B**) root dry weight. Values are shown in the form mean ± SD (*n* = 6). Within each variety, values marked by the same letter do not differ significantly (*p <* 0.05) from one another.

There was no varietal effect with respect to the response to salinity stress, as all four of the varieties exhibited a similar reduction (~50%) in both shoot and root dry weight (Figure 3). P deficiency had a comparable effect on CMS 8704, CMS 8501, and CHH 103 plants: Their shoot dry weight fell to 77.6–78.9% of the performance of non-stressed plants, while their root dry weight fell to 54.7–60.5%. Likewise, maize cultivars CMS 8501, CHH 101, and CHH 103 exhibited a similar response to combined salt and P stress: Their shoot dry weight fell to ~80% of the control, and their root dry weight to ~70%. The effect of combined salt and P stress on CMS 8704 plants was more drastic: Their accumulation of shoot and root dry matter was reduced by, respectively, 92% and 82% (Figure 3). The variety that best adapted to P deficiency was CHH 101: These plants suffered a 69% reduction in shoot dry weight and a 48% reduction in root dry weight (Figure 3).

### 3.2. The Effect of the Stress Treatments on the Concentration of Cations and P

The effect of the various treatments on tissue concentrations of K^+^, Na^+^, and P in plants of each of the four maize varieties is summarized in Table 2.

An analysis of variance revealed that both the treatment and variety main effects for each of the three elements assayed were significant (*p* ≤ 0.001), as were the treatment x variety interactions. As expected, all four maize varieties experienced a significant rise (*p* < 0.05; Tukey HSD test) in the Na^+^ concentration of both the shoot and root in response to both, salinity and combined salt and P stress treatments, but not to P deficiency. Meanwhile, the concentration of K^+^ in both the shoot and root was significantly decreased in each of the varieties by all three stress treatments, with combined salt and P stress inducing the most pronounced decline. Salinity stress reduced the K^+^ concentrations of both the shoot and root more severely than did P deficiency. The application of stress reduced the K^+^/ Na^+^ ratio in both the shoot and root in each of the varieties, but particularly so in response to salinity and combined salt and P stress. Finally, the shoot and root P concentration was significantly reduced in each of the varieties in response to treatments of P deficiency and combined salt and P stress, but not to salinity (Table 2).

### 3.3. Inoculation with PGPR Improved the Performance of Plants Subjected to both Salinity Stress and P Deficiency

**Figure 4:**
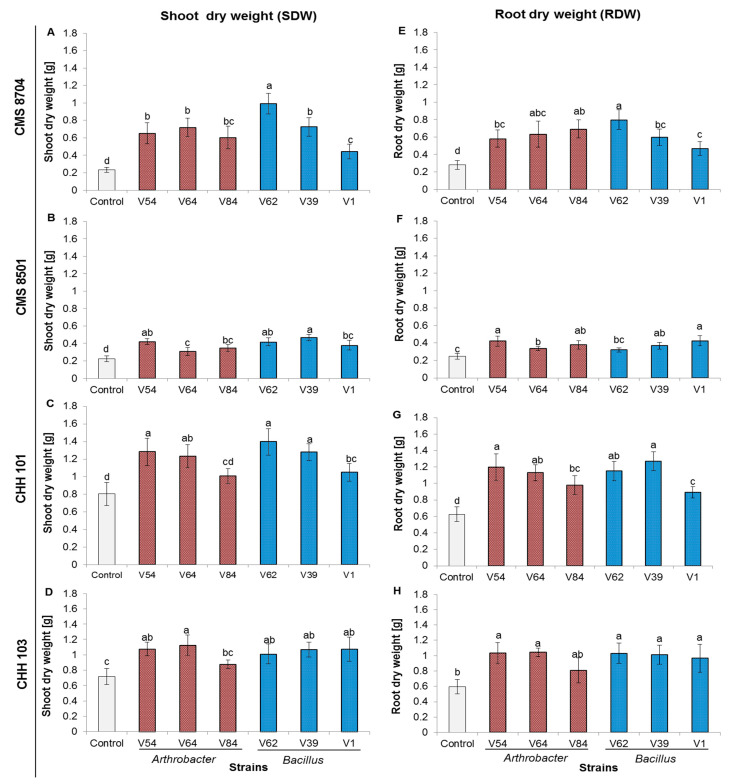
Inoculation with a growth-promoting rhizobacteria (PGPR) promotes the shoot and root growth of maize plants subjected to simultaneous P deficiency and salinity stress. The dry weight of (**A–D**) the shoots, (**E–H**) the roots of plants inoculated with each of the six PGPR strains or non-inoculated. (A,E) CMS 8704, (B,F) CMS 8501, (C,G) CHH 101, (D, H) CHH 103. Measurements were performed six weeks after transplanting in a greenhouse. Values are shown in the form mean ± SD (*n* = 6). Within each variety, values marked by the same letter do not differ significantly (*p <* 0.05) from one another.

Inoculation with each of the six PGPR strains on plants subjected to combined salt and P stress significantly (*p* < 0.05; Tukey HSD test) enhanced the accumulation of biomass by both the shoot and the roots (Figure 4), and also promoted shoot and root elongation (Figure S2). For the composite variety CMS 8704, inoculation with strain V62 was the most effective in promoting shoot and root dry weight (Figure 4A,E), while for CMS 8501, the most effective strain for shoot growth was V39 and for root growth V1 (Figure 4B,F). For CHH 101, the optimal strains for shoot and root growth were, respectively, V62 (Figure 4C) and V39 (Figure 4G), while for CHH 103, strain V64 was the most effective for both shoot (Figure 4D) and root (Figure 4H) growth. Inoculation with each of the six PGPR strains significantly boosted (*p* < 0.05; Tukey HSD test) biomass accumulation by plants of each of the varieties subjected to the stress treatment, although strains V84 and V1 performed less well than the other four (Figure S3). These two strains are the ones compiling the lowest number of PGP traits compared to the other four herein tested strains [11].

**Figure 5:**
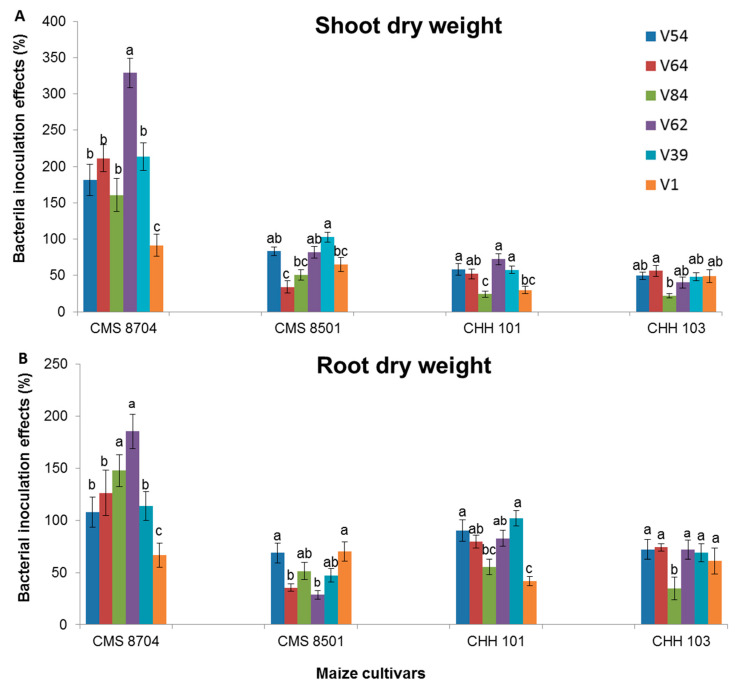
The growth-promoting effect of PGPR inoculation in plants subjected to simultaneous P deficiency and salinity stress. The % improvement in plant performance over non-inoculated control plants with respect to (**A**) shoot dry weight, (**B**) root dry weight of the two composite maize varieties CMS 8704 and CMS 8501, and the two F1 hybrid maize varieties CHH 101 and CHH 103. Measurements were performed six weeks after transplanting in a greenhouse. Values are shown in the form mean ± SD (*n* = 6). Within each variety, values marked by the same letter do not differ significantly (*p <* 0.05) from one another. Control: Non-inoculated plants; V54, V64, and V84: *Arthrobacter* spp. strains, V62, V39, and V1: *Bacillus* spp. strains.

There was a significant level of variation both between the varieties as to the extent of the benefit of inoculation with a given strain, and between the extent of the boost to growth provided by each bacterial strain for a given variety. The advantage of inoculation was generally higher for the composite varieties (particularly CMS 8704) than for the F1 hybrid varieties (Figure 5). The performance of each variety averaged over all six bacterial strains was consistent with this conclusion (Figure S4). CMS 8704 responded most strongly to inoculation with a PGPR in terms of biomass accumulation (Figure 5). The *Arthrobacter* sp. strains V54 and V64, and the *Bacillus* sp. strains V39 and V62 were generally more effective as growth promoters than either *Arthrobacter* sp. strain V84 or *Bacillus* sp. strain V1 (Figure S5).

### 3.4. The Effect of PGPR Inoculation on the Concentration of Cations and P

Plants of each of the four test varieties exposed to combined salt and P stress accumulated less Na^+^ in their biomass when inoculated with a PGPR than those which had not been inoculated (Table 3 and Figure S6). While the presence of each of the bacterial strains moderated the uptake of Na^+^ into the shoot of all four varieties, this effect was not so clear for the roots. A comparison of the shoot and root concentrations of K^+^ and their K^+^/Na^+^ ratios showed that the presence of PGPR was beneficial (Table 3 and Figure S6), but the extent of the benefit was dependent on the choice of both the PGPR strain and the maize variety.

**Figure 6:**
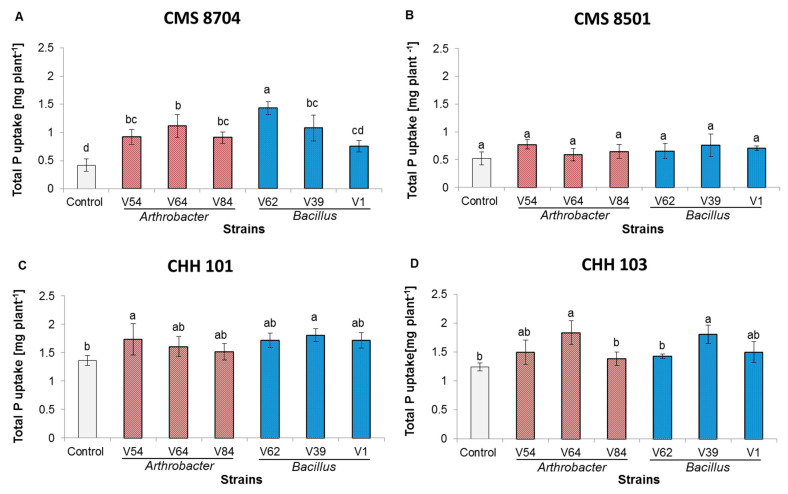
The uptake of P by each of the four test maize varieties was subjected to simultaneous P deficiency and salinity stress and inoculation with PGPR. The improvement in performance over non-inoculated control plants by plants of variety (**A**) CMS 8704, (**B**) CMS 8501, (**C**) CHH 101, (**D**) CHH 103. Measurements were performed six weeks after transplanting in a greenhouse. Values are shown in the form mean ± SD (*n* = 6). Within each variety, values marked by the same letter do not differ significantly (*p <* 0.05) from one another. Control: Non-inoculated plants; V54, V64, and V84: *Arthrobacter* sp. strains, V62, V39, and V1: *Bacillus* sp. strains.

Inoculation also influenced the shoot and root P content (Figure 6). A significant positive effect on CMS 8704 plants was apparent for the five PGPR strains V39, V54, V62, V64, and V84, on CHH 101 plants for two strains (V39 and V54) and on CHH 103 plants for two strains (V39 and V64). The P uptake by CMS 8501 plants was not promoted by PGPR inoculation.

### 3.5. Production by the PGPR of phytohormones

As summarized in Table 4, all six bacterial strains produced some IAA when provided with tryptophan; the amount ranged from 243 (V39) to 6702 (V84) ng mL^−1^. The *Arthrobacter* sp. strains were more productive than the *Bacillus* sp. ones. All six strains were also generated CKs, ABA, and a range of GAs. GA_1_, GA_3_, GA_4_, GA_5_, GA_7_, GA_8_, GA_9_, GA_13_, GA_15_, GA_19_, GA_20_, GA_24_, GA_29_, GA_34_, GA_44_, GA_51_, and GA_53_ were all detected in the culture medium (Figure S7). There were significant differences (p < 0.05) in the individual capability of PGPRs in producing the various GAs: Only GA_1_, GA_8_, and GA_24_ (highlighted in red color) were produced by all six strains, while GA_5_ and GA_7_ (highlighted in yellow) were only produced by the *Bacillus* sp. strains (Figure S7). Among the biologically active GA species (GA_1_, GA_3_, GA_4_, and GA_7_) the most effective producer was V62, which generated 9.1 ng mL^−1^ GA_1_ and 17.5 ng mL^−1^ GA_7_, while strains V1 and V64, respectively, produced the most GA_3_ and the most GA_4_ (Table 4).

## 4. Discussion

Given the well-established heterosis-related advantage of F1 hybrid maize varieties over open-pollinated ones [20], it was not unexpected that a comparison between composite and F1 hybrid varieties would reveal that in the absence of stress, the growth of the latter was superior to that of the former. Less predictable in advance was their relative performance when challenged by salinity stress and/or P deficiency. The vegetative growth of all four varieties was negatively impacted by the imposition of salinity stress (Figure 3), while the F1 hybrid variety CHH 101 coped better than the others under conditions of P deficiency. The impact of P deficiency was more serious than that of salinity stress, as has been noted elsewhere not just for maize [21], but also for both barley [22] and wheat [2]. When the plants were simultaneously exposed to both salinity stress and P deficiency, the resulting growth reduction was more severe than exposure to either P deficiency or salinity stress on their own, a result which contrasts with the outcome of similar experiments conducted in barley, wheat, and maize, where the response to the combined stress regime was reported to be similar to what was induced by P deficiency on its own [2,21,22]. A possible reason for this apparent discrepancy is that the present experiments applied a more intense level of salinity stress (EC of 12 vs. 8–10 ds m^−1^). However, as noted elsewhere, differential responses to abiotic stress can be variety-specific [23]. The growth of the shoot was more markedly compromised by P deficiency than by salinity stress, as would have been predicted, given that shoot growth is generally more sensitive to nutrient deficiency (including not just P, but also nitrogen and iron) than to salinity [21,24]. The observed boost to root elongation in each of the four maize varieties induced by P deficiency mirrors the response to this stress of rice plants [25], reflecting an adaptive response in which the P uptake is enhanced by expanding the surface area of the root system [25,26].

The imposition of salinity stress inevitably increased the tissue Na^+^ content in each of the test varieties. It is well established that the uptake of Na^+^ not only competitively inhibits the uptake of the essential cations K^+^, Ca^2+^, and Mg^2+^ but also interferes with the acquisition and utilization of both P and nitrogen [27]. This ion stress is exacerbated by the greater difficulty plants growing in saline soils experience in acquiring water, thereby compromising their hydration status. For the most part, tissue Na^+^ contents were higher in plants exposed to the simultaneous salinity stress and P deficiency treatment than in those exposed to just salinity stress, a result which conflicts with the behavior of both barley and wheat [2,22]. Both the tissue K^+^ content and K^+^/Na^+^ ratio in both the shoot and root were downgraded by each of the three stress treatments, as was similarly observed for two Australian wheat varieties “Jandorai” and “Janz” [2].

Both the P deficient treatment and the combined salinity stress and P deficient treatment induced a significant decrease in the accumulation of P in the shoot and root of all four maize varieties, as also occurs in rice [28]. Unexpectedly, this was not the case in plants subjected to salinity stress. Exposing groundnut plants to salinity similarly has little effect on their uptake of P [29]. However, salinity stress has been shown to reduce tissue P concentration throughout the plant in both lettuce [30] and wheat [2], while the stress tends to raise shoot and root P concentration in both barley grass and barley [23]. Thus, the suggestion is that the interaction between salinity and P nutrition depends to a large extent on the identity of the plant (with respect to both species and variety), its developmental stage, the growing environment, the intensity of the salinity stress imposed, and the availability of P in the soil [31].

Bacteria, including species within the genera *Arthrobacter* and *Bacillus,* with an ability to solubilize phosphate and/or to mitigate against salinity stress, have been successfully employed to improve the growth of both groundnut [7] and tomato (unpublished data). The present study has demonstrated for the first time that the growth of maize plants simultaneously challenged with salinity stress and P deficiency can be supported by providing the PGPR species as inoculum. The benefit of this inoculation was not consistent across all the maize varieties tested, nor was it independent of which bacterial strain was used. Of the four test varieties, which are genetically quite diverse, the composite variety CMS 8704 proved to be most sensitive to the combined stress treatment, and also responded most strongly to the provision of PGPR. A similar varietal effect has also been noted in rice [32]. With respect to the variation in efficacy among the PGPR strains, the inoculation of CMS 8704 with the *Bacillu*s sp. strain V62 was particularly effective in enhancing growth as measured by all of the parameters, whereas the extent of its benefit to the other three varieties was less uniform. Variation in the ability of a *Bacillus* sp. to promote the growth of lima bean (*Phaseolus lunatus*) varieties has been reported elsewhere [33,34]. Plants able to tolerate salinity stress need to either limit their uptake of Na^+^ and/or increase that of K^+^ [27]. In line with these findings, plants inoculated with strains of both *Arthrobacter* sp. and *Bacillus* sp. had lower shoot Na^+^ concentrations than the non-inoculated control plants in all maize cultivars. In addition, except for *Bacillus* strain V1 in the root K^+^/Na^+^ ratio, all bacterial strains resulted in increased K^+^ concentration and K^+^/Na^+^ ratio in CMS 8704, as compared to their respective non-inoculated control plants. Inoculation of maize with two strains of *Azotobacter* sp. has also been reported to help the plants cope with salinity stress by decreasing their uptake of Na^+^ and increasing that of K^+^ [35]. Similarly, the use of a *Bacillus* sp. inoculum reportedly reduces the accumulation of Na^+^ and increases the K^+^/Na^+^ ratio in both the shoot and root of white clover (*Trifolium repens*) plants subjected to salinity stress [36].

Inoculation with each of the PGPR strains enhanced the uptake of P in three of the four test varieties (the exception was CMS 8501). Strain V39 increased the P uptake of CMS 8704 by 154.5%, that of CHH 101 by 32.5%, and that of CHH 103 by 45.5%. Recent data suggest that inoculation of cucumber plants with *Bacillus subtilis* increases their P uptake by 40% [37]. The likelihood is that the success of PGPR in promoting the P uptake is due to their ability to solubilize unavailable P (such as RP) present in the rhizosphere. Although none of the inocula were able to promote the P uptake by CMS 8501 plants, inoculation was still effective in enhancing their vegetative growth. Similarly, in canola, inoculation with various species of phosphate-solubilizing bacteria succeeded in promoting plant growth in the absence of any evidence that it boosted the P uptake [38].

It has been suggested that an alternative mechanism whereby PGPR stimulate plant growth under stressful conditions could be through their production of one or more of the phytohormones GAs, auxins, CKs or ABA [39]. An analysis of the medium supporting the in vitro growth of the present set of PGPR revealed that they all produced representatives of each of these phytohormones. The auxin IAA has been identified as a catabolite generated by various strains of both *Arthrobacter* sp. and *Bacillus* sp. isolated from the soil surrounding the roots of both maize and wheat [40]. The presence of IAA-producing microorganisms has been shown to stimulate root growth, thereby both expanding the plants’ ability to both extract nutrients from the soil and mitigating against salinity stress [7]. Certain soil bacteria are known to synthesize ABA, a phytohormone intimately related to the plant response to salinity stress [41]. Others, shown to produce CKs, are thought to exert a positive effect on root development [42]. In a recent study, *Bacillus amyloliquefaciens* associated with rice (*Oryza sativa* L.) synthesized a varied amount of gibberellins such as GA_20_, GA_36_, GA_24_, GA_4_, GA_53_, GA_5_, and GA_8_ [43]. Likewise, we observed that the different bacterial strains produced various gibberellins (GA_1_, GA_3_, GA_4_, GA_5_, GA_7_, GA_8_, GA_9_, GA_13_, GA_15_, GA_19_, GA_20_, GA_24_, GA_29_, GA_34_, GA_44_, GA_51_, and GA_53_). Although it has been demonstrated that *Arthrobacter* are able to produce GA [44] this is the first report to show a wide range of GAs produced by this genus.

The growth of the maize plant requires a consistent supply of assimilable nitrogen. While *Arthrobacter* sp. strain V64 reveals lower salinity tolerance and *Bacillus* sp. strain V39 has similar plant growth-promoting potential, both, additionally, have been shown the potential to convert atmospheric nitrogen into a form which can be taken up by plants [11], and so had been predicted as likely to exert a positive effect on plant growth. However, of the six strains, the one responsible for the greatest enhancement on plant growth was *Bacillus* sp. strain V62, which had not been identified as able to fix atmospheric nitrogen. Rather, it was a particularly efficient solubilizer of P, was able to tolerate high salt concentration, and produced significant higher amounts of GA; thus, it appears that improving plant performance under conditions of P deficiency and/or salinity stress requires the ability of the PGPR to solubilize P, even under salt stress, and/or to generate GA, rather than being able to fix nitrogen. The same conclusion has been reached regarding the interaction of *Bacillus amyloliquefaciens* with a range of plant species such as Chinese cabbage, radish, tomato, and mustard plants [45]. To the best of our knowledge, the present study is the first to report the contribution of *Arthrobacter* sp*. and Bacillus* sp. to the growth of maize crops under combined salt + P stress through the solubilization of phosphate and the production of phytohormones. In general, the result of the present study rebuts our hypothesis that bacterial growth-promoting effects will be higher in hybrids than composites cultivars, implying that further work is crucial for understanding such specific plant-bacteria interactions.

## 5. Conclusions

The present experiments have demonstrated that the inoculation of maize with strains of both *Arthrobacter* sp. and *Bacillus* sp. can alleviate the stress imposed by soil salinity and P deficiency, although the extent of the benefit appears to be dependent on the identity of both the maize variety and the bacterial strain. The benefits of inoculation included both improving the supply of mineral nutrients to the plant and promoting its growth through the production of phytohormones. The suggestion is that the choice of the genotype of the maize host and the identity of the PGPR strain together determine the extent of the benefit gained by deliberate inoculation, which is considered to be part of an overall strategy aiming to improve the sustainability of cereal crop production.

## Figures and Tables

**Table 1:** Agronomic characteristics of the four test maize varieties.

Variety Name	Variety Type	Yield (t/ha)	Cycle	Germination Rate	Year of Release	Percentage of Adoption	Grain Properties
**CMS 8704**	Composite	4–6	110–120	94. 5%	1987	9%	yellow, resistant to foliar disease and sweet taste, open pollinated
**CMS 8501**	Composite	4–6	110–120	90%	1985	8%	white, resistant to foliar disease, and open pollinated
**CHH 101**	Hybrid	7–10	110–120	85%	1994	-	yellow and white, high yield
**CHH 103**	Hybrid	7–10	110–120	80%	1994	-	white yield and high yield

Source: Agricultural Research Institute for Development (IRAD).

**Table 2:** The effect of exposure to salinity stress, P deficiency, and salinity stress + P deficiency on the accumulation in the shoot and root of four maize varieties of the cations K^+^ and Na^+^, on the K^+^/Na^+^ ratio, and on the uptake of P. Measurements were taken six weeks after transplanting in a greenhouse.

Maize Cultivars	Growth Conditions	Shoot				Root			
		(mg g^−1^)			K^+^/Na^+^ Ratio	(mg g^−1^)			K^+^/Na^+^ Ratio
		Na^+^	K^+^	P		Na^+^	K^+^	P	
**CMS 8704**	No stress	0.6 ± 0.3 ^c^	79.2 ± 2.0 ^a^	8.5 ± 1.5 ^a^	150.7 ± 61.2 ^a^	7.5 ± 4.2 ^c^	56.9 ± 3.2 ^a^	4.5 ± 0.7 ^a^	9.2 ± 4.2 ^a^
	salt	10.0 ± 0.8 ^b^	65.1 ± 2.4 ^b^	8.8 ± 0.7 ^a^	6.5 ± 1.3 ^c^	35.9 ± 4.2 ^a^	27.0 ± 2.1 ^b^	5.5 ± 1.2 ^a^	0.8 ± 0.1 ^b^
	P stress	1.3 ± 0.1 ^c^	72.3 ± 2.2 ^ab^	0.8 ± 0.02 ^b^	55.1 ± 6.7 ^b^	8.5 ± 1.9 ^c^	42.5 ± 2.7 ^c^	0.9 ± 0.1 ^b^	5.2 ± 1.2 ^ab^
	P + salt stress	35.0 ± 1.0 ^a^	33.3 ± 1.4 ^c^	0.7 ± 0.1 ^b^	1.0 ± 0.03 ^c^	26.9 ± 3.1^b^	13.6 ± 1.5 ^d^	0.7 ± 0.03 ^b^	0.5 ±0.02 ^b^

**CMS 8501**	No stress	0.5 ± 0.07 ^c^	75.0 ± 2.8 ^a^	8.2 ± 2.0 ^a^	133.5 ± 13.1 ^a^	6.8 ± 0.1 ^b^	53.3 ± 5.0 ^a^	4.6 ± 0.9 ^a^	7.7 ± 0.8 ^a^
	salt	11.5 ± 2.5 ^b^	59.3 ± 2.5 ^b^	8.7 ± 0.6 ^a^	5.3 ± 1.0 ^c^	26.1 ± 3.0 ^a^	19.5 ± 0.5 ^c^	3.7 ± 1.0 ^a^	0.7 ± 0.09 ^c^
	P stress	2.9 ± 1.9 ^c^	71.4 ± 3.2^a^	1.4 ± 0.4 ^b^	34.2 ± 14.1^b^	7.6 ± 1.5 ^b^	34.2 ± 3.1 ^b^	1.3 ± 0.1 ^b^	4.6 ± 1.3 ^b^
	P + salt stress	36.6 ± 1.9 ^a^	49.4 ± 1.7 ^c^	0.8 ± 0.01 ^b^	1.3 ± 0.06 ^c^	27.7 ± 0.7 ^a^	16.2 ± 1.2 ^c^	0.9 ± 0.08 ^b^	0.6 ± 0.05 ^c^

**CHH 101**	No stress	0.7 ± 0.2 ^c^	81.1 ± 1.7 ^a^	7.2 ± 0.9 ^a^	125.0 ± 48.6 ^a^	5.9 ± 0.4 ^c^	51.2 ± 2.3 ^a^	3.3 ± 0.3 ^a^	8.7 ± 0.6 ^a^
	salt	7.2 ± 1.0 ^b^	59.9 ± 4.5 ^b^	6.5 ± 0.3 ^a^	8.5 ± 1.6 ^c^	26.7 ± 3.3 ^b^	20.9 ± 1.4 ^c^	2.9 ± 0.3 ^a^	0.8 ± 0.08 ^c^
	P stress	1.6 ± 0.3 ^c^	64.3 ± 1.5 ^b^	1.3 ± 0.1 ^b^	40.6 ± 9.4 ^b^	9.4 ± 3.0 ^c^	36.1 ± 2.5 ^b^	1.0 ± 0.1 ^b^	4.3 ± 1.7 ^b^
	P + salt stress	28.1 ± 5.2 ^a^	42.23 ± 2.2 ^c^	0.8 ± 0.01 ^b^	1.5 ± 0.2 ^c^	31.2 ± 5.0 ^a^	19 ± 1.4 ^c^	0.7 ± 0.05 ^b^	0.6 ± 0.08 ^c^

**CHH 103**	No stress	0.6 ± 0.1 ^c^	75.0 ± 3.6 ^a^	7.2 ± 0.9 ^a^	123.0 ± 17.7 ^a^	5.9 ± 1.1 ^c^	48.6 ± 2.1 ^a^	3.5 ± 0.6 ^a^	8.6 ± 2.3 ^a^
	salt	10.8 ± 3.2 ^b^	65.2 ± 2.0 ^b^	8.5 ± 1.5 ^a^	6.4 ± 1.5 ^c^	28.4 ± 5.5 ^b^	26.9 ± 2.0 ^c^	4.5 ± 0.6 ^a^	0.9 ± 0.2 ^c^
	P stress	1.0 ± 0.3 ^c^	66.7 ± 2.0 ^b^	0.8 ± 0.04 ^b^	71.8 ± 21.4 ^b^	7.1 ± 0.8 ^c^	40.4 ± 4.6 ^b^	0.9 ± 0.07 ^b^	5.7 ± 1.0 ^b^
	P + salt stress	24.9 ± 3.0 ^a^	35.3 ± 1.2 ^c^	0.6 ± 0.03 ^b^	1.4 ± 0.1 ^c^	37.9 ± 1.8 ^a^	16.7 ± 2.2 ^d^	0.7 ± 0.06 ^b^	0.4 ± 0.07 ^c^

Values are shown in the form mean ± SD (*n* = 6). Within each variety, values marked by the same letter do not differ significantly (*p <* 0.05) from one another.

**Table 3:** The effect of PGPR inoculation on the accumulation of cations (K^+^, Na^+^), the K^+^/Na^+^ ratio, and the uptake of P in the shoot and root of the four maize varieties in plants exposed to both salinity stress and P deficiency. Measurements were performed six weeks after transplanting in a greenhouse.

Maize Cultivars	Bacterial Treatments	Shoot				Root			
		(mg g^−1^)			K^+^/Na^+^ Ratio	(mg g^−1^)			K^+^/Na^+^ Ratio
		K^+^	Na^+^	P		K^+^	Na^+^	P	
	Control	33.3 ± 1.4 ^b^	35.0 ± 1.1 ^a^	0.6 ± 0.1 ^b^	0.9 ± 0.03 ^c^	13.6 ± 1.5 ^c^	26.9 ± 3.1 ^a^	0.7 ± 0.03 ^b^	0.5 ± 0.02 ^b^
CMS 87 04	V54	53.6 ± 2.2 ^a^	23.4 ± 1.3 ^b^	0.8 ± 0.01 ^a^	2.2 ± 0.1 ^ab^	21.9 ± 1.2 ^ab^	25.6 ± 2.7 ^ab^	0.9 ± 0.1 ^ab^	0.8 ± 0.09 ^ab^
	V64	49.8 ± 4.3 ^a^	22.0 ± 2.9 ^b^	0.8 ± 0.08 ^a^	2.3 ± 0.5 ^ab^	24.4 ± 3.8 ^a^	25.0 ± 1.0 ^ab^	1.0 ± 0.09 ^a^	0.9 ± 0.1 ^a^
	V84	50.8 ± 5.5 ^a^	23.5 ± 1.8 ^b^	0.8 ± 0.03 ^a^	2.1 ± 0.2 ^ab^	23.7 ± 2.6 ^a^	27.0 ± 6.2 ^a^	0.8 ± 0.05 ^ab^	0.9 ± 0.3 ^a^
	V62	57.2 ± 3.9 ^a^	21.7± 2.6 ^b^	0.9 ± 0.02 ^a^	2.6 ± 0.2 ^a^	22.9 ± 2.2 ^a^	24.7 ± 2.1 ^ab^	1.0 ± 0.05 ^a^	0.9 ± 0.02 ^a^
	V39	52.1 ± 4.5 ^a^	26.5 ± 2.9 ^b^	0.8 ± 0.07 ^ab^	1.9 ± 0.2 ^ab^	17.2 ± 2.4 ^bc^	28.5 ± 5.3 ^a^	1.0 ± 0.1 ^a^	0.6 ± 0.1 ^b^
	V1	51.1 ± 3.2 ^a^	28.2 ± 5.3 ^ab^	0.9 ± 0.04 ^a^	1.8 ± 0.4 ^ab^	15.7 ± 1.7 ^c^	18.2 ± 2.1 ^b^	1.0 ± 0.06 ^a^	0.8 ± 0.3 ^ab^
CMS 8501	Control	49.4 ± 1.7 ^ab^	36.6 ± 1.9 ^a^	0.8 ± 0.01 ^a^	1.3 ± 0.06 ^c^	16.2 ± 1.2 ^b^	27.7 ± 0.7 ^a^	0.9 ± 0.08 ^a^	0.5 ± 0.05 ^c^
	V54	55.7 ± 1.5 ^ab^	21.1 ± 2.9 ^bc^	0.8 ± 0.07 ^a^	2.6 ± 0.4 ^a^	31.7 ± 5.3 ^a^	24.9 ± 2.3 ^ab^	1.1 ± 0.1 ^a^	1.2 ± 0.09 ^a^
	V64	59.6 ± 3.6 ^a^	24.4 ± 2.1 ^bc^	0.9 ± 0.05 ^a^	2.4 ± 0.17 ^ab^	23.8 ± 4.4 ^ab^	23.2 ± 0.9 ^b^	1 ± 0.05 ^a^	1.0 ± 0.1 ^b^
	V84	60.9 ± 0.7 ^a^	22.7 ± 0.3 ^bc^	0.7 ± 0.08 ^a^	2.6 ± 0.06 ^a^	16.4 ± 1.4 ^b^	21.4 ± 3.5 ^b^	1.1 ± 0.09 ^a^	0.7 ± 0.06 ^c^
	V62	56.7 ± 2.5 ^ab^	19.3 ± 2.2 ^c^	0.9 ± 0.06 ^a^	2.9 ± 0.2 ^a^	16.2 ± 1.8 ^b^	27.4 ± 1.2 ^a^	0.9 ± 0.01 ^a^	0.5 ± 0.04 ^c^
	V39	49.7 ± 7.2 ^ab^	26.5 ± 2.0 ^b^	0.8 ± 0.06 ^a^	1.8 ± 0.3 ^bc^	18.5 ± 0.8 ^b^	23.6 ± 1.1 ^ab^	0.9 ± 0.1 ^a^	0.7 ± 0.001 ^c^
	V1	45.8 ± 7.4 ^b^	24.8 ± 2.5 ^bc^	0.9 ± 0.04 ^a^	1.8 ± 0.1 ^bc^	18.2 ± 1.1 ^b^	25.7 ± 3.0 ^ab^	1.0 ± 0.07 ^a^	0.7 ± 0.04 ^c^
CHH 101	Control	42.2 ± 2.2 ^a^	28.1 ± 5.2 ^a^	0.8 ± 0.01 ^b^	1.5 ± 0.2 ^a^	19 ± 1.4 ^a^	31.2 ± 5.0 ^a^	0.7 ± 0.05 ^ab^	0.6 ± 0.08 ^a^
	V54	46.1 ± 2.6 ^a^	19.0 ± 1.2 ^b^	0.79 ± 0.05 ^b^	2.4 ± 0.2 ^a^	22.7 ± 2.6 ^a^	31.8 ± 4.0 ^a^	0.7 ± 0.09 ^ab^	0.7 ± 0.1 ^a^
	V64	49.4 ± 4.8 ^a^	18.4 ± 6.3 ^b^	0.7 ± 0.04 ^b^	2.9 ± 1.2 ^a^	21.1 ± 2.2 ^a^	26.7 ± 2.0 ^a^	0.6 ± 0.04 ^b^	0.7 ± 0.07 ^a^
	V84	47.1 ± 1.1 ^a^	20.1 ± 3.3 ^ab^	0.8 ± 0.05 ^b^	2.3 ± 0.4 ^a^	22.7 ± 1.0 ^a^	29.0 ± 3.6 ^a^	0.8 ± 0.08 ^ab^	0.7 ± 0.1 ^a^
	V62	50.0 ± 3.7 ^a^	18.8 ± 2.5 ^b^	0.7 ± 0.05 ^b^	2.7 ± 0.5 ^a^	22.4 ± 2.0 ^a^	29.4 ± 2 ^a^	0.7 ± 0.05 ^ab^	0.7 ± 0.1 ^a^
	V39	48.7 ± 4.5 ^a^	18.3 ± 1.7 ^b^	0.7 ± 0.03 ^b^	2.6 ± 0.4 ^a^	22.1 ± 1.0 ^a^	28.6 ± 1.8 ^a^	0.8 ± 0.05 ^ab^	0.7 ± 0.08 ^a^
	V1	49.8 ± 3.3 ^a^	21.9 ± 3.1^ab^	1.1 ± 0.1 ^a^	2.3 ± 0.3 ^a^	21.6 ± 1.3 ^a^	28.7 ± 3.6 ^a^	0.8 ± 0.03 ^a^	0.7 ± 0.1 ^a^
CHH 103	Control	35.2 ± 1.2 ^c^	24.9 ± 3.0 ^a^	0.6 ± 0.03 ^d^	1.4 ± 0.1 ^c^	16.7 ± 2.2 ^c^	37.9 ± 1.8 ^ab^	0.7 ± 0.06 ^c^	0.4 ± 0.07 ^b^
	V54	44.8 ± 4.1 ^b^	20.3 ± 1.0 ^ab^	0.7 ± 0.03 ^c^	2.2 ± 0.2 ^bc^	23.3 ± 3.1 ^ab^	28.0 ± 5.5 ^b^	0.8 ± 0.03 ^abc^	0.8 ± 0.3 ^a^
	V64	44.8 ± 3.8 ^b^	15.4 ± 3.1 ^bc^	0.9 ± 0.03 ^a^	2.9 ± 0.3 ^ab^	20.0 ± 1.2 ^bc^	30.6 ± 5.3 ^ab^	1.0 ± 0.1 ^a^	0.6 ± 0.1 ^ab^
	V84	47.1 ± 3.7 ^ab^	13.6 ± 0.9 ^c^	0.8 ± 0.008 ^b^	3.4 ± 0.4 ^a^	21.3 ± 2.6 ^abc^	29.8 ± 4.4 ^ab^	0.9 ± 0.08 ^ab^	0.7 ± 0.1 ^ab^
	V62	44.6 ± 2.8 ^b^	13.6 ± 2.8 ^c^	0.7 ± 0.009 ^cd^	3.4 ± 0.9 ^a^	22.2 ± 2.6 ^abc^	30.9 ± 2.6 ^ab^	0.7 ± 0.01 ^bc^	0.72 ± 0.1 ^ab^
	V39	44.4 ± 3.6 ^b^	14.0 ± 1.9 ^c^	0.9 ± 0.07 ^a^	3.2 ± 0.6 ^ab^	26.6 ± 2.6 ^a^	27.9 ± 1.6 ^b^	0.9 ± 0.07 ^ab^	0.9 ± 0.09 ^a^
	V1	53.1 ± 1.5 ^a^	17.8 ± 1.3 ^bc^	0.7 ± 0.02 ^cd^	2.9 ± 0.2 ^ab^	23.8 ± 3.5 ^ab^	23.7 ± 2.7 ^a^	0.8 ± 0.05 ^abc^	1.0 ± 0.2 ^a^

Values are shown in the form mean ± SD (*n* = 6). Within each variety, values marked by the same letter do not differ significantly (*p <* 0.05) from one another. Control: Non-inoculated plants; V54, V64, and V84: *Arthrobacter* sp. strains, V62, V39, and V1: *Bacillus* sp. strains.

**Table 4:** The production of phytohormones by the six PGPR strains when cultured in vitro.

		Phytohormone Production						
		(ng g^−1^) (ng mL^−1^)						
Bacterial Isolates		Abscisic Acid (ABA)	Auxin (IAA)	Cytokinins (CKs)	Gibberellic Acid (GA)			
					GA_1_	GA_3_	GA_4_	GA_7_
**Arthrobacter strains**	V54	1.1 ± 0.2 ^a^	4781.1 ± 397.2 ^b^	38.3 ± 5.2 ^ab^	3.7 ± 0.2 ^b^	nd	0.22 ± 0.04 ^b^	0.01 ± 0.003 ^b^
	V64	1.9 ± 0.3 ^a^	5169.8 ± 223.7 ^b^	27.1 ± 2.7 ^c^	0.1 ± 0.02 ^d^	nd	4.9 ± 0.4 ^a^	nd
	V84	1.3 ± 0.3 ^a^	6702.2 ± 253.0 ^a^	23.3 ± 1.1 ^c^	2.0 ± 0.2 ^c^	0.01 ± 0.002 ^b^	nd	nd
**Bacillus strains**	V62	2.0 ± 0.1 ^a^	385.6 ± 22.6 ^c^	29.2 ± 2.1 ^bc^	9.1 ± 0.1 ^a^	0.03 ± 0.01 ^b^	4.2 ± 1.1 ^a^	17.5 ± 0.6 ^a^
	V39	1.2 ± 0.6 ^a^	242.9 ± 50.1 ^c^	42.6 ± 6.2 ^a^	3.8 ± 0.7 ^b^	0.07 ± 0.04 ^b^	0.3 ± 0.02 ^b^	0.1 ± 0.008 ^b^
	V1	1.4 ± 0.1 ^a^	279.8 ± 15.3 ^c^	47.7 ± 1.3 ^a^	0.9 ±0.1^d^	0.16 ± 0.04 ^a^	0.6 ± 0.08 ^b^	0.17 ± 0.08 ^b^

Values are shown in the form mean ± SD (*n* = 3). Within each variety, values marked by the same letter do not differ significantly (*p <* 0.05) from one another. ND: Not detected.

## Supplementary Materials

The following are available online at https://www.mdpi.com/2076-2607/8/7/1005/s1.
