# Supplementary material for: The Response of Maize to Inoculation with Arthrobacter sp. and Bacillus sp. in Phosphorus-Deficient, Salinity-Affected Soil
Source: Microorganisms. 2020 Jul 4;8(7):1005. doi: 10.3390/microorganisms8071005 (PMC7409341; doi:10.3390/microorganisms8071005)
Supplement: Supplementary file 1 [file microorganisms-08-01005-s001.pdf]

The response of maize to inoculation with *Arthrobacter* sp. and *Bacillus* sp. in phosphorus-deficient, salinity-affected soil

Tchuisseu Tchakounté Gylaine Vanissa [1, 5, 6*], Beatrice Berger [2], Sascha Patz [3], Matthias Becker [2], Veronika Turečková [4], Ondřej Novák [4], Danuše Tarkowská [4], Fankem Henri [5], Silke Ruppel [1]

[1] Leibniz Institute of Vegetable and Ornamental Crops Grossbeeren, Theodor- Echtermeyer-Weg 1, 14979 Grossbeeren, Germany

[2] Institute for National and International Plant Health, Julius Kuehn-Institute – Federal Research Centre for Cultivated Plants, Messeweg 11/12, 38104, Braunschweig, Germany

[3] Algorithms in Bioinformatics, Center for Bioinformatics, University of Tuebingen, Sand 14, 72076 Tuebingen, Germany

[4] Laboratory of Growth Regulators, The Czech Academy of Sciences, Institute of Experimental Botany and Palacky University, Slechtitelu 27, CZ-783 71 Olomouc, Czech Republic

[5] Department of Plant Biology, Faculty of Sciences, University of Douala, P.O. Box: 24157 Douala, Cameroon

[6] Faculty of Life sciences Humboldt-University of Berlin, Invalidenstraße 42, 10115 Berlin, Germany

tchuisseu@igzev.de
beatrice.berger@julius-kuehn.de
sascha.patz@uni-tuebingen.de
matthias.becker@julius-kuehn.de
veronika.tureckova@upol.cz
novako@ueb.cas.cz
tarkowska@ueb.cas.cz
fankemhenri@yahoo.fr
ruppel@igzev.de

[*]correspondence: tchuisseu@igzev.de; Tel: +49 (0)33701-78357

Supplementary figures

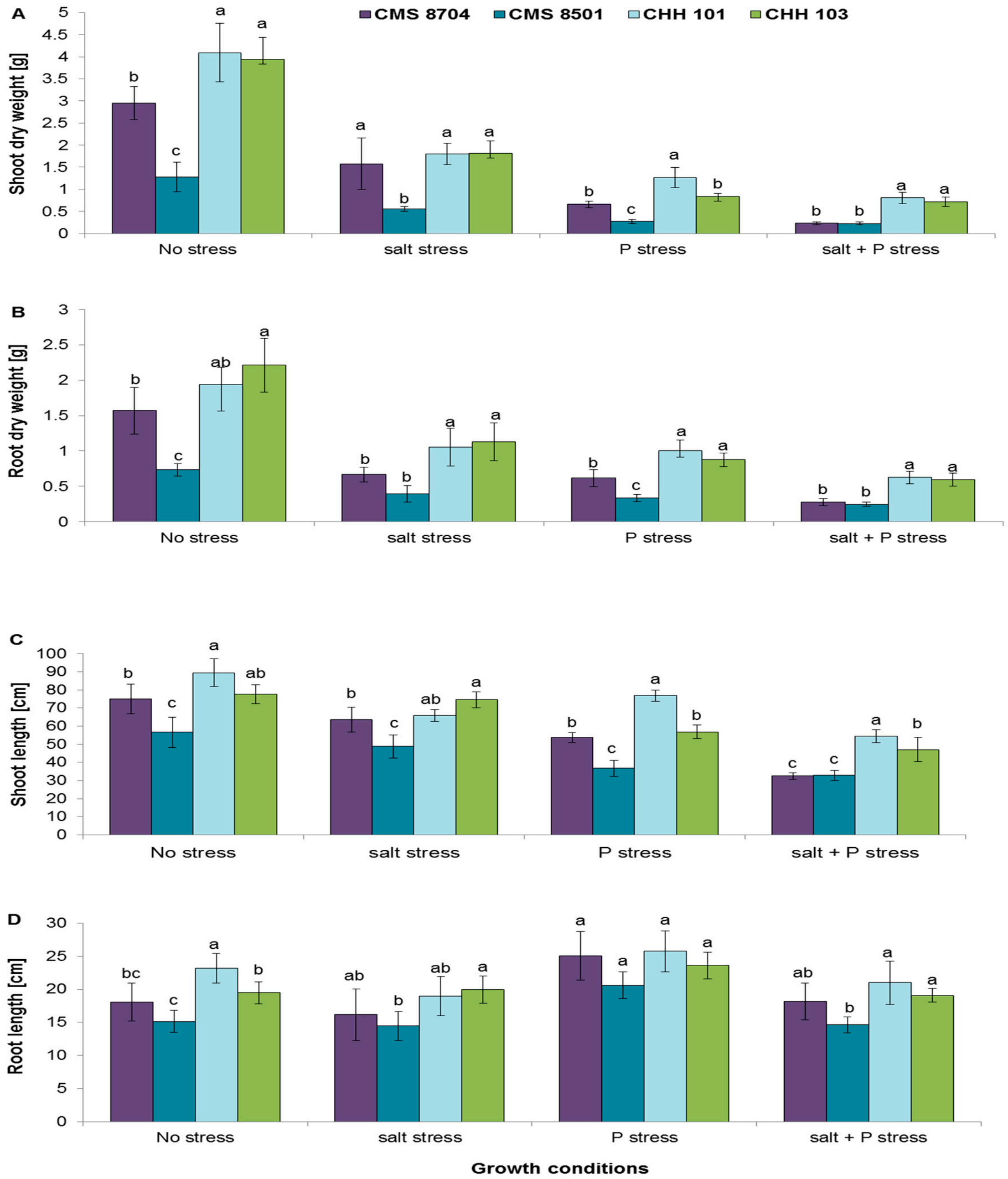

Figure S1. The growth of each of the four test maize varieties (CMS 8704, CMS 8501: composite varieties, CHH 101, CHH 103: F1 hybrid varieties) in response to the four treatments (no stress, salinity stress, P deficiency and salinity stress + P deficiency) with respect to (**A**) shoot dry weight, (**B**) root dry weight, (**C**) shoot length, (**D**) root length. Values shown in the form mean ± SD (n = 6). Within each variety, values marked by the same letter do not differ significantly ($p < 0.05$) from one another.

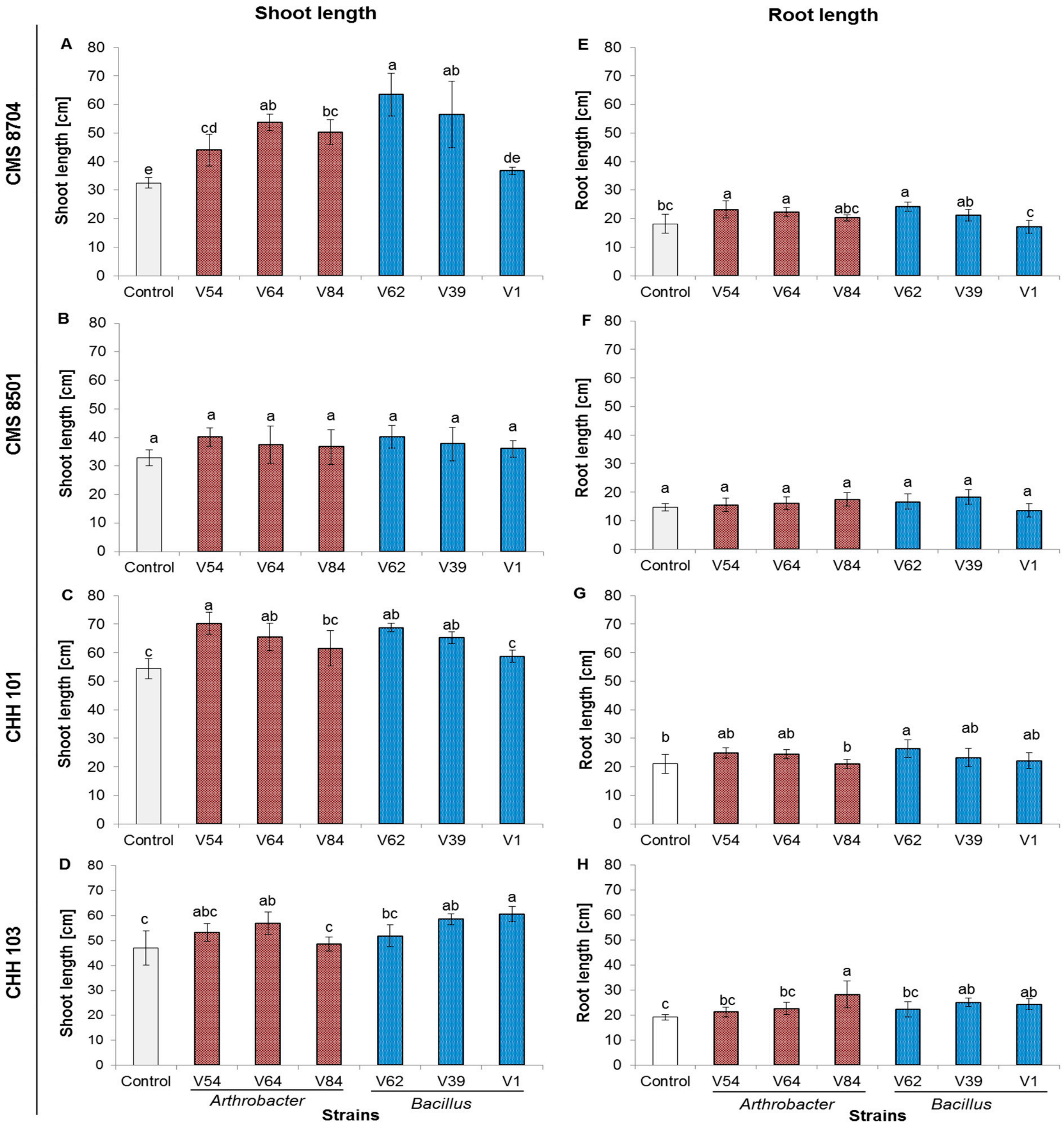

Figure S2. PGPR inoculation promotes the shoot and root growth of maize plants subjected to simultaneous P deficiency and salinity stress. (**A–D**) Shoot length, (**E–H**) root length of (A,E) CMS 8704, (B,F) CMS 8501, (C,G) CHH 101, (D,H) CHH 103. Measurements were performed after a six week growth period in a greenhouse. Values shown in the form mean ± SD ($n = 6$). Within each variety, values marked by the same letter do not differ significantly ($p < 0.05$) from one another. Control: non-inoculated plants; V54, V64 and V84: *Arthrobacter* spp. strains, V62, V39 and V1: *Bacillus* spp. strains.

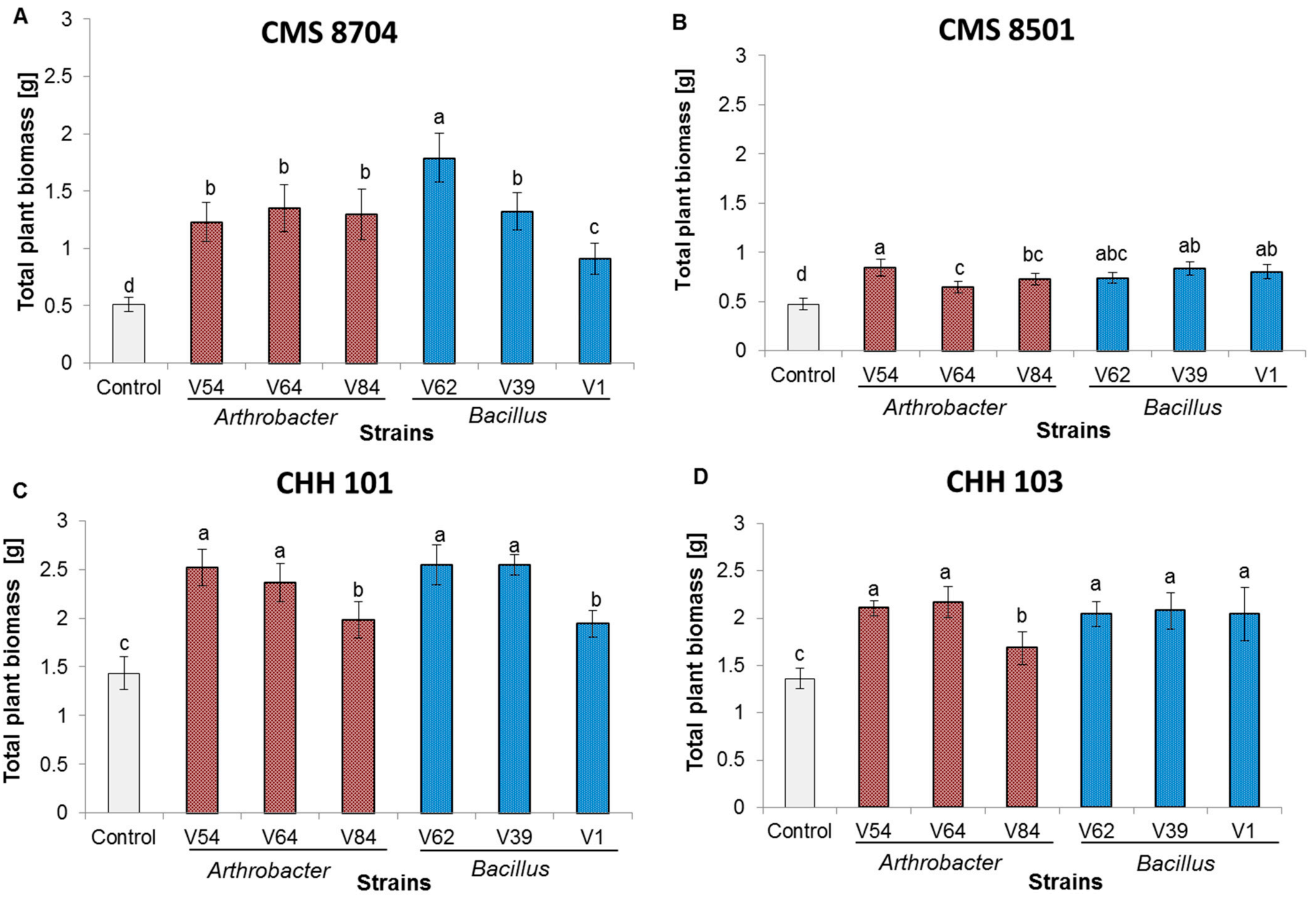

Figure S3. Inoculation with PGPR enhances the biomass accumulation of the four test maize variety plants subjected to simultaneous P deficiency and salinity stress. (**A**) CMS 8704, (**B**) CMS 8509, (**C**) CHH 101, (**D**) CHH 103 inoculated with one of *Arthrobacter* sp. strains V54, V64 or V84, or *Bacillus* sp. strains V62, V39 or V1. Measurements were performed after a six week growth period in a greenhouse after transplanting. Values shown in the form mean ± SD (n = 6). Within each variety, values marked by the same letter do not differ significantly ($p < 0.05$) from one another. Control: non-inoculated plants.

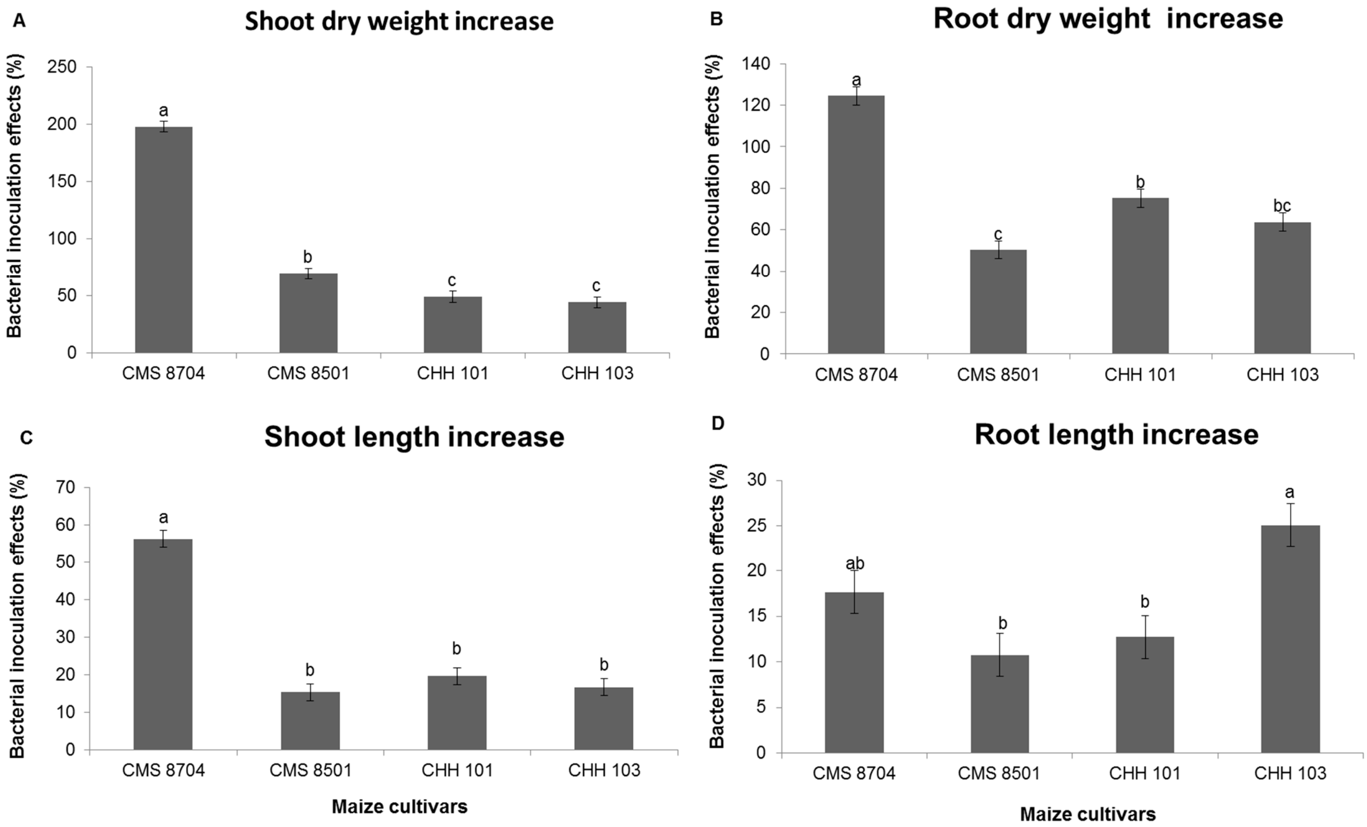

Figure S4. The differential varietal response to inoculation with PGPR. The bars indicate the mean (averaged over all six bacterial strains) increase over non-inoculated plants with respect to (**A**) shoot dry weight, (**B**) root dry weight, (**C**) shoot length, (**D**) root length of the two composite varieties CMS 8704 and CMS 8501, and the two F1 hybrid varieties CHH 101 and CHH 103. The plants were subjected to simultaneous P deficiency and salinity stress and were assessed after a six week growth period in a greenhouse. Values shown in the form mean ± SD (n = 6). Within each trait, values marked by the same letter do not differ significantly ($p < 0.05$) from one another.

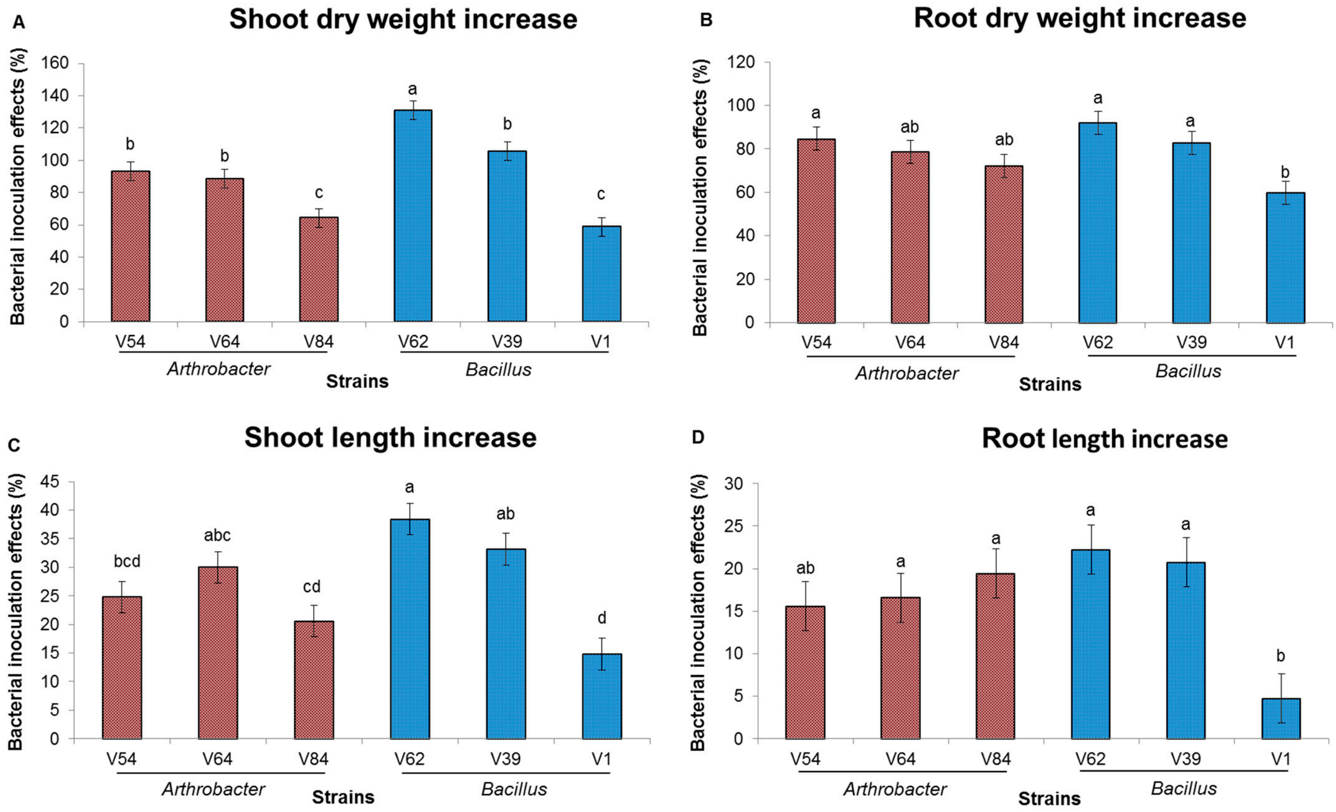

Figure S5. The differential varietal response to inoculation with PGPR. The bars indicate the mean (averaged over all four maize varieties) increase over non-inoculated plants with respect to (**A**) shoot dry weight, (**B**) root dry weight, (**C**) shoot length, (**D**) root length achieved by inoculating with either an *Arthrobacter* sp. strain (V54, V64, V84) or a *Bacillus* sp. strain (V62, V39, V1). The plants were subjected to simultaneous P deficiency and salinity stress and were assessed after a six week growth period in a greenhouse. Values shown in the form mean ± SD (n = 6). Within each trait, values marked by the same letter do not differ significantly ($p < 0.05$) from one another.

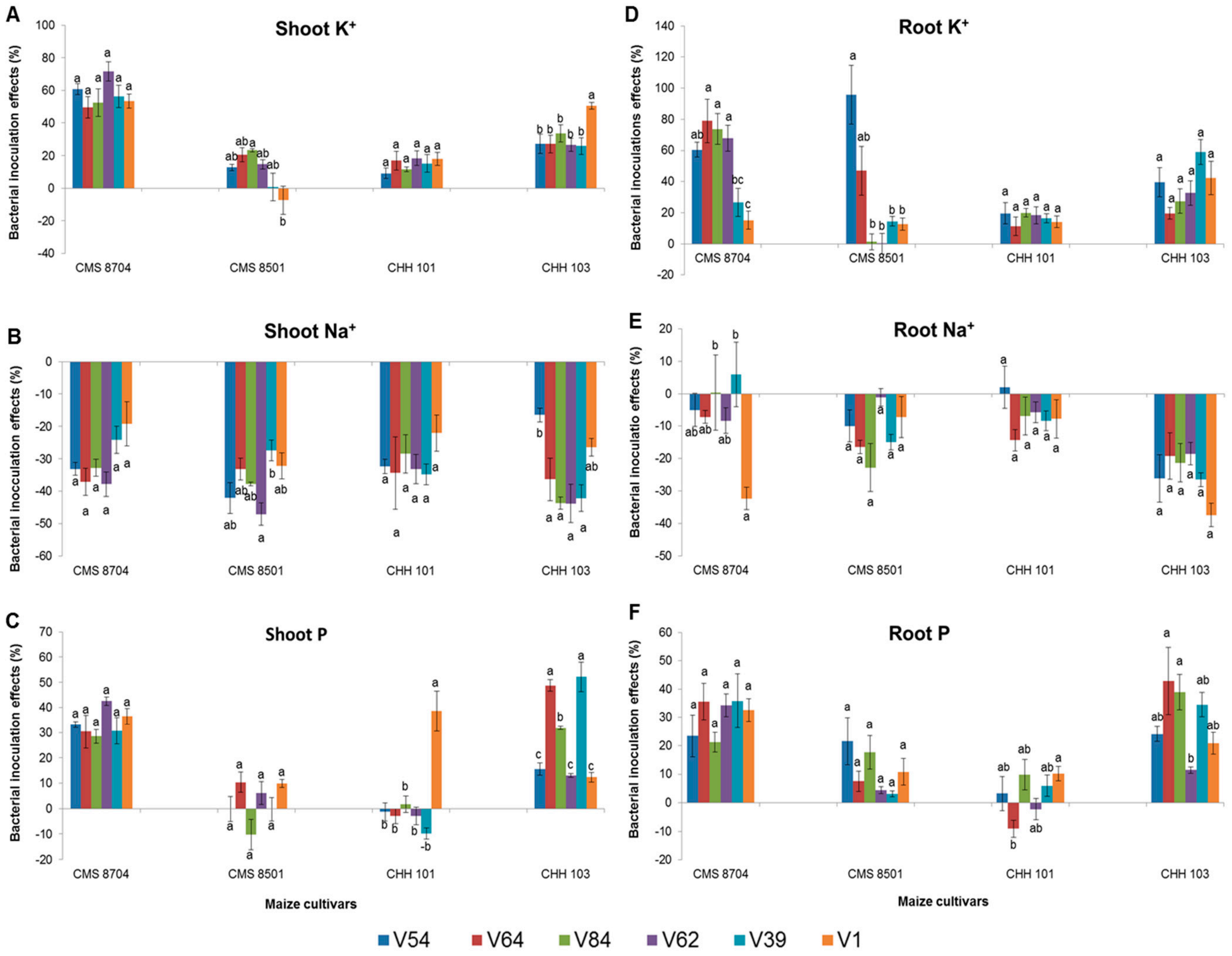

Figure S6. The effect of PGPR inoculation on the concentrations of Na^+, K^+ and P of maize plants subjected to simultaneous P deficiency and salinity stress. The % improvement in performance over non-inoculated control plants shown by each variety (CMS 8704, CMS 8501, CHH 101, CHH 103). The content in the shoot (**A–C**) and root (**D–F**) of (A,D) K^+, (B,E) Na^+, (C,F) P. Measurements were taken six weeks after transplanting in a greenhouse. Values shown in the form mean ± SD (n = 6). Within each variety, values marked by the same letter do not differ significantly ($p < 0.05$) from one another.

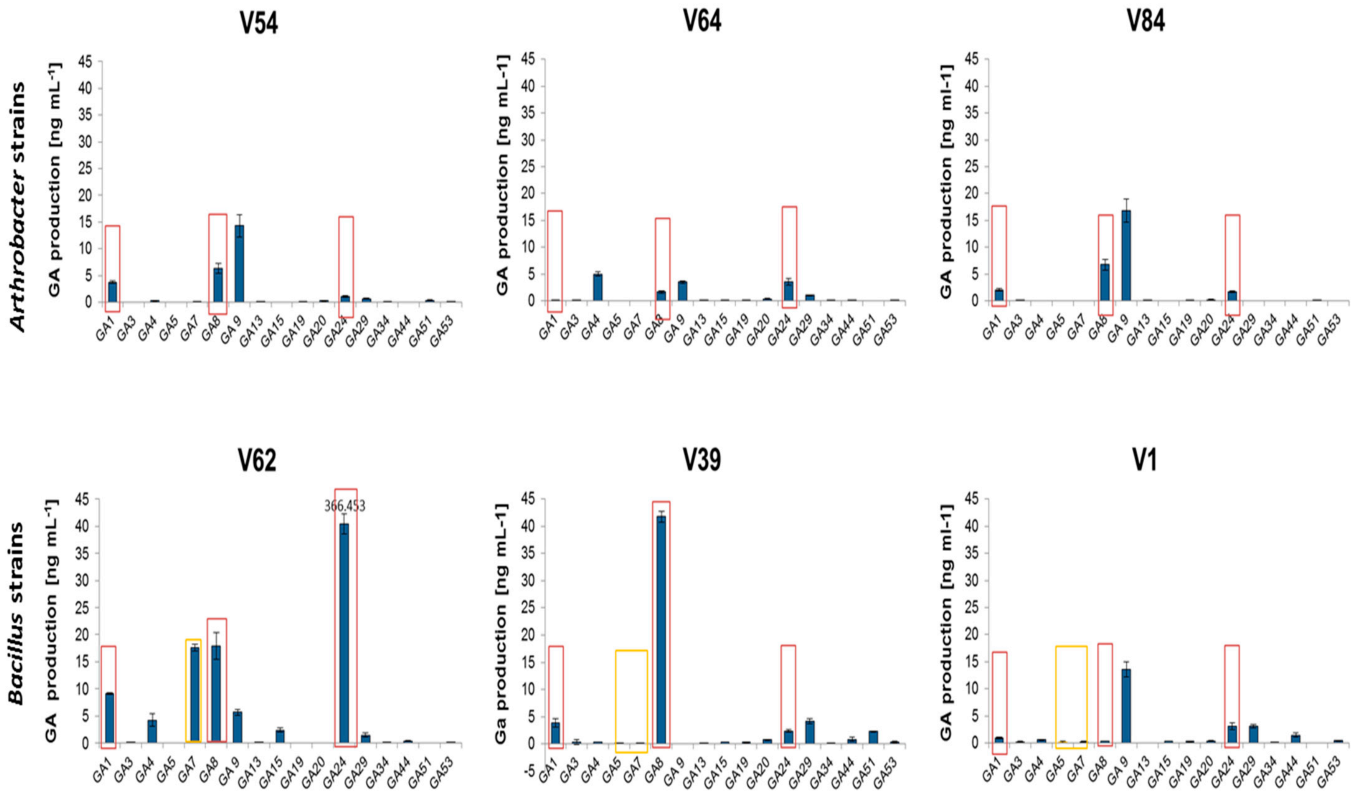

Figure S7. Gibberellins (GAs) in PGPR cells cultured *in vitro.* Compounds highlighted in red were produced by each of the six strains (V54, V64, V84: *Arthrobacter* sp. strains, V62, V39, V1: *Bacillus* sp. strain), while those highlighted in yellow were only produced by the *Bacillus sp.* strains.
